# 546 Obesity’s Impact on Pediatric Scald Burns: How Much Weight Does It Carry?

**DOI:** 10.1093/jbcr/iraf019.175

**Published:** 2025-04-01

**Authors:** Christian Hudson-Bradford, Rachael Galvin, Elika Ridelman, Darina Malinova, Justin Klein, Christina Shanti

**Affiliations:** Department of Surgery, Division of Pediatric Surgery, Children’s Hospital of Michigan/ Wayne State University School of Medicine; Children’s Hospital of Michigan Burn Center; Department of Surgery, Division of Pediatric Surgery, Children’s Hospital of Michigan/ Wayne State University School of Medicine; Wayne State University; Department of Surgery, Division of Pediatric Surgery, Children’s Hospital of Michigan/ Wayne State University School of Medicine; Department of Surgery, Division of Pediatric Surgery, Children’s Hospital of Michigan/ Wayne State University School of Medicine

## Abstract

**Introduction:**

Childhood obesity is a growing public health concern in the United States, affecting about 14.7 million children and adolescents (CDC). There is increasing evidence that obesity affects morbidity and mortality in adults, but less is known about its impact on pediatric patients, particularly those with burn injuries. This study investigates the relationship between obesity and wound-healing outcomes in this population.

**Methods:**

A retrospective chart review was conducted on 389 pediatric patients (1 month - 18 years) admitted to a regional burn center with ≥ 5% TBSA scald burns between July 2015 and January 2024. Patients with staph scalded skin or who received follow-up treatment elsewhere were excluded. Patients were categorized into WFL or BMI groups using the WHO/CDC-adapted growth charts for children < 2 years old (WFL Group) and the 2000 CDC growth charts for children 2-20 years old (BMI Group). The primary outcome was time to complete wound healing. Secondary outcomes included scar management, late grafting (delayed grafting due to poor wound healing), outpatient therapies, and hospitalization. ANOVA tests were utilized to compare healing times between weight groups, and chi-square tests for categorical data.

**Results:**

ANOVA tests revealed no significant differences in healing time between BMI categories in both the 5-group (underweight, healthy weight, overweight, obese, severely obese) and 3-group (underweight, healthy weight, overweight/obese/severely obese combined) analyses, nor within the 3-group WFL categories (underweight, healthy weight, overweight). Chi-square tests for patients < 2 and patients ≥2 revealed no significant associations between WFL/BMI categories and the need for Level 1 (p = 0.1727; p = 0.6422), Level 2 (p = 0.4816; p = 0.7282), or Level 3 (p = 0.6238; p = 0.7932) Scar Management, as well as Late Grafting (p = 0.2236; p = 0.7646). No significant relationship was found between WFL/BMI categories and the need for Grafting During the Index Admission for patients < 2 (p = 0.1807) or ≥2 (p = 0.1477). However, in the combined ≥2 group, a moderate relationship (p = 0.0463) suggests a potential association between higher BMI and grafting in older children.

**Conclusions:**

While childhood obesity is a growing concern, this study suggests it may not significantly affect wound healing time or other outcomes in pediatric scald burn patients. Our findings suggest that obesity may not be a significant independent risk factor for wound-healing complications in this population. However, further investigation is warranted to explore other potential impacts of obesity and other burn types, such as flame burns.

**Applicability of Research to Practice:**

This research could inform clinical practice by potentially alleviating concerns about delayed healing or complications in obese children with scald burns. These findings enhance our understanding of obesity’s impact on pediatric burn injuries, aiding treatment decisions and improving patient outcomes.

**Funding for the Study:**

N/A

